# Secondary polycythaemia from chronic hypoxia is a risk for cerebral thrombosis: a case report

**DOI:** 10.1186/s12883-023-03277-5

**Published:** 2023-06-09

**Authors:** Min Zhou, Xiaoxia Liu, Li Li

**Affiliations:** Department of Neurology, Ningbo No. 2 Hospital, 41 Xi’bei Street, Ningbo, Zhejiang China

**Keywords:** Secondary polycythemia, Hypoxia, Thrombosis, Pathophysiology, Case report

## Abstract

**Background:**

Secondary polycythemia is considered the usual complication of chronic hypoxia. It can theoretically increase the oxygen-carrying capacity, but this adaptive trait has a deleterious effect because the blood viscosity increases, which can induce significant morbidity and mortality, such as stroke and myocardial infarction.

**Case presentation:**

A 55-year-old man with a history of a congenitally small main pulmonary artery presented to the emergency department with sustained unsteady walking, dizziness and vertigo. Evaluation revealed elevated hemoglobin and superior posterior circulation cerebral artery thrombosis. The patient was treated with high flux inhalation of oxygen and anti-platelet aggregation.

**Conclusions:**

The involvement of cerebral vessels has rarely been reported in chronic hypoxia cases. The present case is the first case of superior posterior circulation cerebral artery thrombosis due to chronic hypoxia in a patient with a congenitally small main pulmonary artery. This case demonstrates the importance of recognizing some chronic diseases that can lead to hypoxia and secondary polycythemia thereby leading to hypercoagulable state and subsequent thrombosis.

## Background

Polycythemia, an elevation in the concentration of hemoglobin, can result from increased red cell mass or a reduced plasma volume. An increased red cell mass can be caused by numerous primary (bone marrow) and secondary causes [1]. Multiple factors contribute to the development of secondary polycythemia [2]. Hypobaric hypoxia is the main reason for the change in pathophysiology, and the physiological changes involve oxygen intake, transportation and utilization. This may lead to a significant increase in blood viscosity, causing vessel damage and microcirculatory disturbances, vascular thrombosis, extensive organ damage, sleep disorders, and death. High oxygen affinity hemoglobin should also be considered in the assessment of patients with polycythemia [3]. Here, we report a case of cerebral thrombosis in a man in a hypercoagulable state caused by secondary polycythemia due to chronic hypoxia. This is the first case of superior posterior circulation cerebral artery thrombosis due to chronic hypoxia in a patient with a congenitally small main stem pulmonary artery. Importantly, secondary polycythemia is a known side effect of chronic hypoxia; thus, when some diseases lead to chronic hypoxia in patients with elevated hemoglobin and hematocrit, thrombosis must be considered in the differential diagnosis.

## Case presentation

**Fig. 1:**
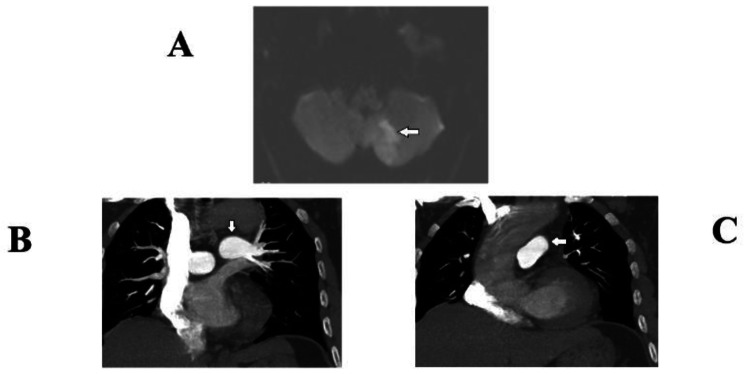
(**A**) Abnormal signal of the left cerebellar dentate nuclei on diffusion-weighted MRI. (**B**) and (**C**) CTA of the pulmonary artery revealed the main pulmonary artery (approximately 18mm in diameter) is smaller than the right and left branched (approximately 19mm in diameter) of pulmonary artery

A 55-year-old man presented to the emergency department of our hospital with an unsteadily gait, his body veering toward the left, dizziness and vertigo. His symptoms started four days before presenting to the emergency department. He was a clerk and did not have physical work, denied any current tobacco use, hormone therapy or recreational drug use. He does not have a history of diabetes, hypertension, previous thromboembolism, atrial fibrillation, sleep apnea, underwater/high altitude sickness or any other cardiac or chronic lung disease. There was no family history of venous/arterial thrombosis, malignancy, or polycythemia. On physical examination, the patient’s blood pressure was 181/120 mmHg, and his oxygen saturation was 85% on a 2 L nasal cannula. The patient had not an arterial puncture before supplementation of oxygen. During the neurological examination, he could not complete a left-sided alternating movement test, a heel-knee-tibia test or a finger-nose test, and he was found to have horizontal nystagmus and a Glasgow Coma scale of 15/15. The patient did not have any other pathological signs. Our clinical diagnosis was stroke. Initial laboratory findings showed polycythemia, a B-hemoglobin level of 20.6 g/dL (11.7–17.4 g/dL), a hematocrit of 0.60 (0.35–0.51), a white cell count of 11.9 × 10^9^/L (3.5–9.5 × 10^9^/L) and a thrombocyte count of 189 × 10^9^/L (125–350 × 10^9^/L). A blood film revealed no abnormal morphology in any of the cell lineages. Further investigation to identify an underlying cause, including codon 617 of Janus Kinase 2 (*JAK2*^*V617F*^) mutation testing and *BCR/ABL* translocation, were negative. Mutations in exon 12 of the JAK2 gene and high-affinity hemoglobin (HAH) were not tested. The levels of serum erythropoietin and lipid analysis were normal. Thrombophilia screening tests, including coagulation factor, antithrombin, protein C levels and protein S levels, factor V Leiden mutation and fibrinolytic activity, were all normal. Antineutrophil antibodies, antiphospholipid antibodies and anti-nuclear antibodies were also negative. No evidence of atherosclerosis was found in the cerebral vascular examinations. Transthoracic echocardiogram and 24-hour Holter monitoring failed to reveal a clear source of the thrombus. He did not have any surgical treatments for fractures, and he did not have any recent venipuncture or a history of underwater diving. Magnetic resonance imaging (MRI) showed thrombosis in the left cerebellar dentate nuclei (Fig. 1A). A computed tomography angiogram (CTA) of the pulmonary artery showed that the main pulmonary artery (mPA) diameter was 18 mm, which was smaller than the branched artery (Fig. 1B C) (the mPA diameter was 25.8 ± 2.9 mm in normal men in China). The final diagnosis of the presented case was cerebral thrombosis caused by secondary polycythemia due to chronic hypoxia.

The patient and his family did not accept the phlebotomy. So the patient was initially treated with high flow oxygen and 200 mg of aspirin daily and then discharged on oxygen therapy. He underwent rehabilitation in a rehabilitation center for 1 month. He was discharged home, and continued treatment with low-dose oral aspirin and oxygen supplementation. In addition, we had discussed with vascular surgeons and they did not recommend performing surgery to correct the pulmonary artery because of uncertain therapeutic efficacy. Meanwhile, our treatment is effective.

The patient was followed up in the clinic 3 months later. His symptoms had resolved completely, and his hematology report revealed a decrease in the B-hemoglobin/hematocrit of 18.9 g/dL/0.56. There has been no recurrence of thrombotic events. He was instructed to continue treatment with 100 mg oral aspirin and oxygen supplementation in the future.

## Discussion

Posterior circulation cerebral artery thromboses may present in several ways depending on the area of ischemia. Dizziness, vertigo, nausea, and vomiting are common initial symptoms. Other signs include ataxia, diplopia, speech abnormalities and changes in mental status [4]. Embolism, atherothrombosis and vertebral artery dissection are the common documented causes of posterior circulation cerebral artery thrombosis [5, 6]. Atherothrombosis is thought to contribute to cerebral artery thrombosis in elderly patients, while it is traditionally thought to arise from a cardiovascular source in younger patients [7]. The cause of thrombosis is either inherited or acquired in patients in prothrombotic states. Although it was easy to make the diagnosis of thrombosis by assessing the typical clinical and imaging manifestations in our patient, the cause of thrombosis was not clear. Other common causes include arteritis, oral contraceptive use, classic myeloproliferative neoplasms, protein C deficiency, moyamoya disease [8], sleep apnea, underwater/high altitude sickness and some unknown factors.

Cerebral arterial and venous thromboembolism is a known complication of polycythemia. The reported incidence of thrombosis in polycythemia vera patients is 12–40% [9]. According to the World Health Organization (WHO) 2016 criteria at time of the diagnostic workup in the patient, a diagnosis of polycythemia vera requires B-hemoglobin and hematocrit thresholds of 16.5 g/dL and 49% in men and 16.0 g/dL and 48% in women, respectively [10]. Secondary polycythemia results from increased erythropoiesis and elevated hematocrit levels, which commonly occur in response to chronic hypoxemia. Acute high-altitude illnesses, chronic obstructive pulmonary disease and obstructive sleep apnea are common causes of hypoxemia, leading to secondary polycythemia.

Our patient had congenitally small main stem pulmonary artery, which led to the development of anoxia. Secondary polycythemia might be a beneficial adaptation to hypoxia, leading to a hypercoagulable state. No mutations related to polycythemia vera was demonstrated. An analysis for the exon12 mutation was not made, but it is most likely that the patient had a secondary polycythaemia. This interpretation is supported that the B-haemoglobin revealed a decrease after 3 months observation. Hypercoagulability has presented in patients with polycythemia [11]. Hypoxemia, platelet activation, erythrocytosis and red cell abnormalities, chronic endothelial damage, high-shear stress of the vessel wall and blood hyperviscosity have been linked to thrombotic complications in patients with hypercoagulability. However, genetic adaptation abrogates hypoxia-induced erythrocytosis, and Tibetans do not exhibit increased B-hemoglobin concentrations at high altitudes [12].

Erythropoietin is essential for erythropoiesis. Thus, the stimulation of erythropoiesis increases the red blood cell count and hemoglobin concentration. When oxygen-depleted blood travels through the kidney, erythropoietin is secreted, increasing the amount of erythrocytes to alleviate the state of acute hypoxia in human body. However, chronic hypoxia leads to excess production of erythrocytes, causing secondary polycythemia, which is characterized by an overproduction of red blood cells and B-hemoglobin causing hyperviscosity and thrombosis [13]. During prolonged hypoxia, hypoxia-inducible factors (HIFs) accelerate erythropoiesis, which upregulates erythropoietin transcription. This results in an increase in red blood cell production and the delivery of more oxygen to the tissues [14]. The red blood cells (RBCs) that are generated during hypoxia are specifically deficient in catalase. In model rats, it was found that the biochemical changes in the RBC membrane and RBC cell contents may independently impair blood flow through the formation of RBC aggregates that have the potential to directly block blood flow in small vessels, which can contribute to ischemia and infarction, especially with respect to cerebral blood flow [15].

It is generally considered that secondary polycythaemia is a benign disorder and thrombotic rates appeared lower than in polycythaemia vera [16], which is a malignant condition, a neoplasm. However, one recent study has reported that the risk of thrombosis in patients with secondary polycythemia resembles that in patients with low-risk polycythemia vera [17], suggesting that in terms of thrombosis, secondary polycythemia may indeed not be as benign as previously reported. Additionally, the diagnosis and management of secondary polycythemia has become increasingly challenging. There are concerns about methods used for investigations and therapeutic interventions. Furthermore, no matter what the erythrocytosis etiology is, age, hypertension, obesity, etc. significantly clustered with thrombosis risk, emphasizing the importance of controlling these in secondary polycythemia patients [18]. From a therapeutic perspective, in order to adjust for the cause, driving the secondary polycythaemia, a target hematocrit is usually 0.50–0.55, and whether phlebotomies and aspirin use are beneficial for secondary polycythemia patients remains controversial [2] and may be nocuous. Finally, additional prospective trials are needed to more comprehensively assess certain issues related to secondary polycythemia, and special considerations and closer follow-ups are needed to potentially decrease the risk of thrombotic complications in secondary polycythemia patients.

## Conclusions

In conclusion, thromboembolic events caused by secondary polycythemia induced by hypoxia from a congenitally small main stem pulmonary artery are rare. It probably is less recognized or remains underreported. It is important to realize that some chronic diseases that can lead to hypoxia may cause secondary polycythemia that can lead to thrombosis.

## Data Availability

N/A.

## Abbreviations

mmHg: Millimeter of mercury
g: Gram
dL: Deciliter
JAK2: Just another kinase2
BCR/ABL: BCR-ABL fusion gene
HAH: High affinity haemoglobin
MRI: Magnetic resonance imaging
CTA: Computed tomography angiogram
mPA: Main pulmonary artery
WHO: World Health Organization
HIF: Hypoxia-inducible factors
RBCs: Red blood cells
